# Antiviral activity of salivary microRNAs for ophthalmic herpes zoster

**DOI:** 10.1186/1742-4682-9-21

**Published:** 2012-06-07

**Authors:** M Kemal Irmak, Uzeyir Erdem, Ayhan Kubar

**Affiliations:** 1 High Council of Science, Gulhane Military Medical Academy, Ankara, Turkey; 2 Department of Ophtalmology, Gulhane Military Medical Academy, Ankara, Turkey; 3 Department of Virology, Gulhane Military Medical Academy, Ankara, Turkey

## Abstract

Ophthalmic herpes zoster is a common ocular infection caused by the varicella-zoster virus (VZV). Viral mRNA transcripts play a major role in the replicative cycle of the virus and current antiviral agents have little effect in preventing and treating the complications. Therapeutic use of saliva for certain painful ocular diseases such as ophthalmic herpes zoster is a well-known public practice in our region. We thought that antiviral activity of saliva may stem from salivary microvesicles and we aimed to look for molecules with antiviral activity in these vesicles. As a possible candidate for antiviral activity, salivary microvesicles contain at least 20 microRNAs (miRNAs), small noncoding RNAs, which suppress the translation of target mRNAs. miRNAs not only participate in maintenance of normal cell functions, but are also involved in host–virus interactions and limit the replication of certain virus types. Thus, miRNA gene therapy by targeting mRNAs required for VZV survival may find a niche in the treatment of ophthalmic herpes zoster. But, how could salivary microvesicles reach into the corneal cells to demonstrate their antiviral activity. We suggest that human salivary microvesicles can be effective carriers of miRNA for corneal cells, because they contain a molecular machinery for vesicle trafficking and fusion allowing them to be endocytosed by target cells. After binding to the plasma membrane, microvesicles seem to enter into the corneal cells through the clathrin-mediated endocytosis. In the cytosol, human salivary miRNAs base-pair with specific viral mRNAs and inhibit their translation, thus limiting the replication of the virus.

## Ophthalmic herpes zoster

Herpes zoster is a common infection caused by the varicella-zoster virus (VZV). Approximately 20% of the world's population suffers from herpes zoster at least once in a lifetime, with 10% to 20% having ophthalmic involvement (ophthalmic herpes zoster) limited mainly to the cornea [1,2]. Ophthalmic herpes zoster has a very variable course; some cases resolve without trace after a minimum of treatment, others become indolent with chronic cellular and lipid infiltration. These patients present with varying degrees of decreased vision, pain, and light sensitivity [3]. Unfortunately this tends to occur more in young people and therefore these lesions should be observed and treated carefully [4]. Viral DNA is mainly found in mononuclear cells, in keratocytes, and in epithelial cells of the cornea [5]. Antiviral agents have demonstrated some success in resolving early signs and symptoms, but they have little effect in preventing and treating late complications [2].

## Replicative cycle of the varicella-zoster virus

Extensive transcription mapping showed that VZV contains 78 different mRNA transcripts of 6.8 kb or less [6]. After the entry of VZV into the cells, early viral mRNA transcripts are produced in the nucleus, translated in the cytoplasm, and proteins they encode are transported back to the nucleus, where they facilitate viral DNA replication [7]. Thereafter, late viral mRNAs are transcribed, translated, and proteins they encode are transported back to the nucleus for assembly into nascent capsids. Newly replicated DNA is then packaged into capsids in the nucleus, enveloped in the cytosol and transported to the cytoplasmic membrane, where the virions are released [7].

## Salivary miRNAs as antiviral agents

Therapeutic use of saliva for certain painful ocular diseases such as ophthalmic herpes zoster is a well-known public practice in our region. We thought that antiviral activity of saliva may stem from salivary microvesicles (see Ref. [8] for details of microvesicles) and we aimed to look for molecules with antiviral activity in these microvesicles. As a possible candidate for antiviral activity, salivary microvesicles contain at least 20 miRNAs, small noncoding RNAs, which suppress the translation of target mRNAs [9]. List of the most highly expressed human microRNAs in salivary microvesicles are: hsa-let-7b, hsa-let-7c*, hsa-miR-128, hsa-miR-150*, hsa-miR-17, hsa-miR-1908, hsa-miR-212, hsa-miR-27b*, hsa-miR-29b, hsa-miR-29c, hsa-miR-335, hsa-miR-379*, hsa-miR-433, hsa-miR-454, hsa-miR-483-3p, hsa-miR-584, hsa-miR-621, hsa-miR-652, hsa-miR-760 and hsa-miR-888* [9]. More recently, it was shown that salivary microvesicles also contain GW182, which is required for miRNA function [10].

MicroRNAs (miRNAs) are short non-coding RNAs that bind to and repress complementary mRNA targets [11]. The human genome contains more than 500 miRNAs, and each miRNA can repress hundreds of genes by targeting specific regions in the mRNA transcripts [12].

An important feature of microRNAs is their remarkable stability and resistance to degradation, especially compared to mRNA [9]. Increasing evidence suggests that miRNAs not only participate in maintenance of normal cell functions, but are also involved in host–virus interactions and limit the replication of certain virus types [13-17]. Thus, miRNA molecules that alter the function of specific viral mRNA transcripts represent a new strategy for treating viral diseases [18], and miRNA gene therapy by targeting mRNAs required for VZV survival may find a niche in the treatment of ophthalmic herpes zoster.

Since virus genome-specific non-coding small RNAs were reported to serve as successful antiviral agents against a number of ocular viral infections [19-21], we suggest here that miRNAs from salivary microvesicles can be used as therapeutic agents for ophthalmic herpes zoster. The transfer of miRNAs to the infective cornea through the salivary microvesicles and their subsequent regulation could result in the amelioration of the clinical manifestations of this ocular disease. But, how could these microvesicles reach into the corneal cells to demonstrate their antiviral activity.

Topical delivery to the eye is the most convenient way of ocular miRNA delivery, since it offers a noninvasive, simple delivery strategy with large surface absorption area [22]. However, the implementation of this type of therapy is actually hampered by the lack of an efficient carrier [23,24]. Therefore, a safe and effective miRNA delivery system is needed for miRNA-based antiviral therapy to achieve significant benefits in clinical settings. We suggest here that human salivary microvesicles can be effective carriers of miRNA for corneal cells, because they contain a molecular machinery for vesicle trafficking and fusion allowing them to be endocytosed by target cells. Human salivary microvesicles contain about 500 different proteins, a significant portion of which is involved in endocytosis, vesicular trafficking and fusion [25]. Of these, clathrin and Rab proteins (Rab5) are implicated in the process of endocytosis and engaged in transport and fusion [26]. In particular, Rab5 mediates the capture and fusion of clathrin-coated vesicles with endosomal membranes [27]. Other proteins like heat shock protein 70 participate in membrane translocation and decoating [25]. In addition to proteins, human salivary microvesicles contain more than 500 mRNA transcripts which could be transferred to other cells, translated into proteins and modulate the gene expression at their new location [28].

## Mechanisms of the microvesicle endocytosis by corneal cells

Membrane-bound compartments newly formed from the corneal cell surface normally enter the endosomal/lysosomal network, which is an inhospitable environment [29]. Viruses which are valuable models of cellular entry and intracellular trafficking pathways avoid lysosomal degradation, which is a dead end for many particles in a classic endocytic pathway. Entry of adenovirus into the corneal cells is emerging as a useful paradigm in the field. After adenovirus is internalized through clathrin-mediated endocytosis; it is entrapped in the endosomes [30]. Protein VI of the adenovirus causes membrane disintegration and allows adenovirus to escape the endosomes by forming pores [31-34]. In cytosol, heat shock protein 70 facilitates the disassembly of the coat protein from the virus (decoating of adenovirus) [35]. Similarly salivary microvesicles contain a molecular machinery for clathrin mediated endocytosis and are capable to decoat the microvesicular membrane by heat shock protein 70 [25]. Therefore, after cellular uptake, these microvesicles are able to harbor a mechanism that mimics that used by adenoviral particles to escape from the endosomal/lysosomal pathway and proceed to the cytosol.

## A scenario for the therapy of ophthalmic herpes zoster through microvesicular miRNA of saliva

After binding to the plasma membrane, microvesicles enter the corneal cells through the clathrin-mediated endocytosis. After penetration into the cell, microvesicles translocate Rab5 proteins to the outer surface of the vacuolar membrane by a syringe-like mechanism while moving toward the endosome [36]. These proteins help the microvesicles to pass from membrane to the endosome and vacuoles containing microvesicles fuse with endosome. In the endosome, some other proteins take role for the microvesicles to escape into the cytosol [37]. In cytosol, decoating of the microvesicular membrane occurs with the help of heat shock protein 70 and the released mRNA is translocated into the cytosol directly together with miRNA. In the cytosol, the linear copy of the microvesicular mRNA is translated into proteins by the cellular enzymes. In this way, about 500 transcripts representing the microvesicular transcriptome from saliva can be expressed in the corneal cells [28]. Human salivary microvesicles also express more than 20 different miRNAs with a potential to repress the viral mRNA transcripts directly [9]. These miRNAs base-pair with specific viral mRNAs and inhibit their translation, thus limiting the replication of the virus. Since a single miRNA can act to repress many complementary viral mRNAs, this method may allow saliva to be a useful tool in the treatment of the ophthalmic herpes zoster [38].

## Competing interests

The authors declared that they have no competing interest.

## Authors’ contributions

MKI proposed the concept of antiviral activity of salivary microRNAs for ocular diseases and reviewed the relevant literature. UE was primarily responsible for the consistency of ophthalmic herpes zoster with the hypothesis. AK investigated the entry of adenovirus into the corneal cells. All authors read and agreed the final manuscript.
